# Supplementary material for: Higher Synovial Immunohistochemistry Reactivity of IL-17A, Dkk1, and TGF-β1 in Patients with Early Psoriatic Arthritis and Rheumatoid Arthritis Could Predict the Use of Biologics
Source: Biomedicines. 2024 Apr 8;12(4):815. doi: 10.3390/biomedicines12040815 (PMC11048598; doi:10.3390/biomedicines12040815)
Supplement: Supplementary file 1 [file biomedicines-12-00815-s001.zip › biomedicines-2891751-Supplementary.pdf]

Supplementary Table 1.- The logistic regression analysis of the dependent variable (use of biologics).

Variables for the use of biologics

	B	ET	Wald	gl	Sig.	Exp(B)	95,0% IC for Exp(B)	
							Lower	Upper
Male	2.059	0.832	6.119	1	0.013	7.840	1.534	40.076
Age	-0.016	0.017	0.834	1	0.361	0.984	0.952	1.018
csDMARD	0.900	0.739	1.484	1	0.223	2.460	0.578	10.473
erosive	0.624	0.968	0.416	1	0.519	1.867	0.280	12.444

Age, sex, baseline use of csDMARDs, and erosive radiographic damage were selected as independent variables.
